# Supplementary material for: An Ex Vivo 3D Tumor Microenvironment-Mimicry Culture to Study TAM Modulation of Cancer Immunotherapy
Source: Cells. 2022 May 8;11(9):1583. doi: 10.3390/cells11091583 (PMC9101510; doi:10.3390/cells11091583)
Supplement: Supplementary file 1 [file cells-11-01583-s001.zip › cells-1674000-supplementary.pdf]

Supplemental Information

An Ex Vivo 3D Tumor Microenvironment-Mimicry Culture to Study TAM Modulation of Cancer Immunotherapy

Yan-Ruide Li, Yanqi Yu, Adam Kramer, Ryan Hon, Matthew Wilson, James Brown, and Lili Yang

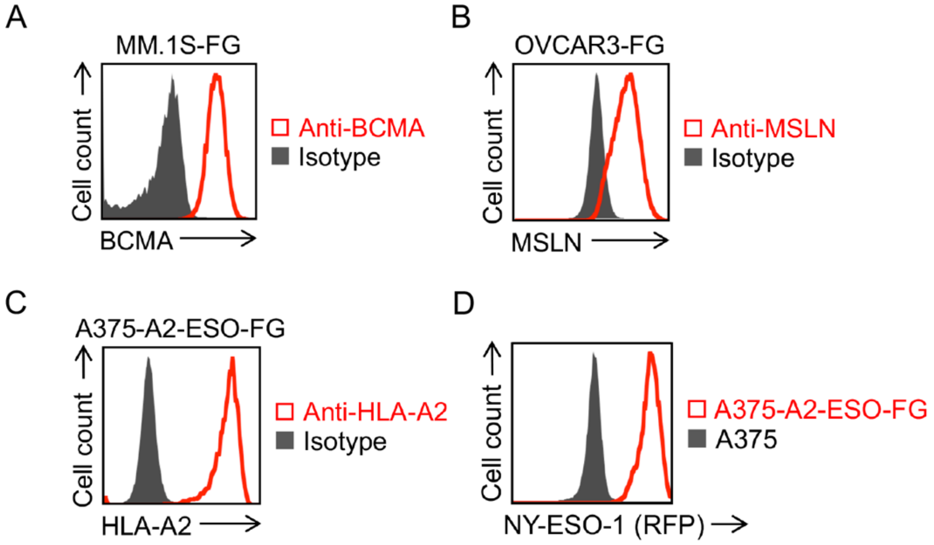

Figure S1. Generation of three tumor cell lines. Related to Figure 3.

(A) FACS plots showing the detection of BCMA on MM.1S-FG cells.

(B) FACS plots showing the detection of mesothelin on OVCAR3-FG cells. MSLN, mesothelin.

(C) FACS plots showing the detection of HLA-A2 on A375-A2-ESO-FG cells.

(D) FACS plots showing the detection of NY-ESO-1 tumor antigen (indicated by RFP) on A375-A2-ESO-FG cells. The parental A375 cells were included as a staining control.

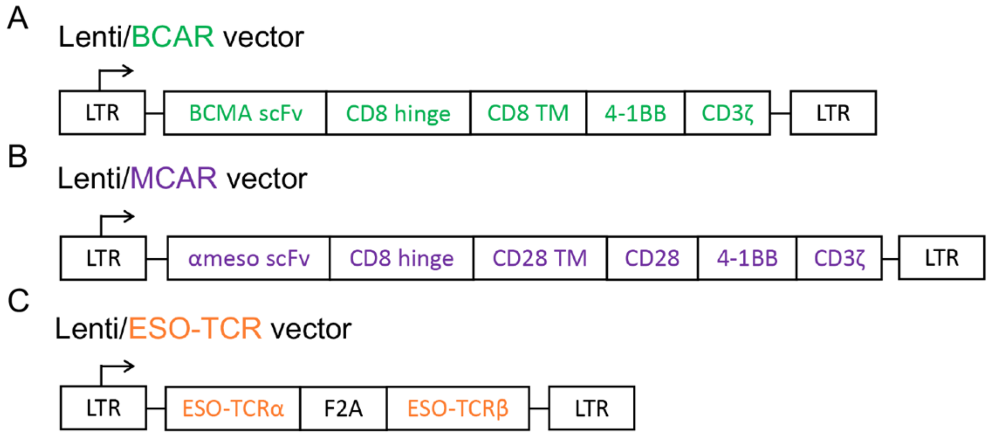

Figure S2. Schematic of Lenti/BCAR (A), Lenti/MCAR (B), and Lenti/ESO-TCR (C) vectors. Related to Figure 3.
Lenti/BCAR, lentiviral vector encoding a BCMA CAR gene; Lenti/MCAR, lentiviral vector encoding a mesothelin CAR gene; Lenti/ESO-TCR, lentiviral vector encoding HLA-A2-restricted, NY-ESO-1 tumor antigen-specific human CD8 TCR gene; LTR, long terminal repeat; BCMA, B-cell maturation antigen; scFv, single-chain variable fragment; TM, transmembrane.

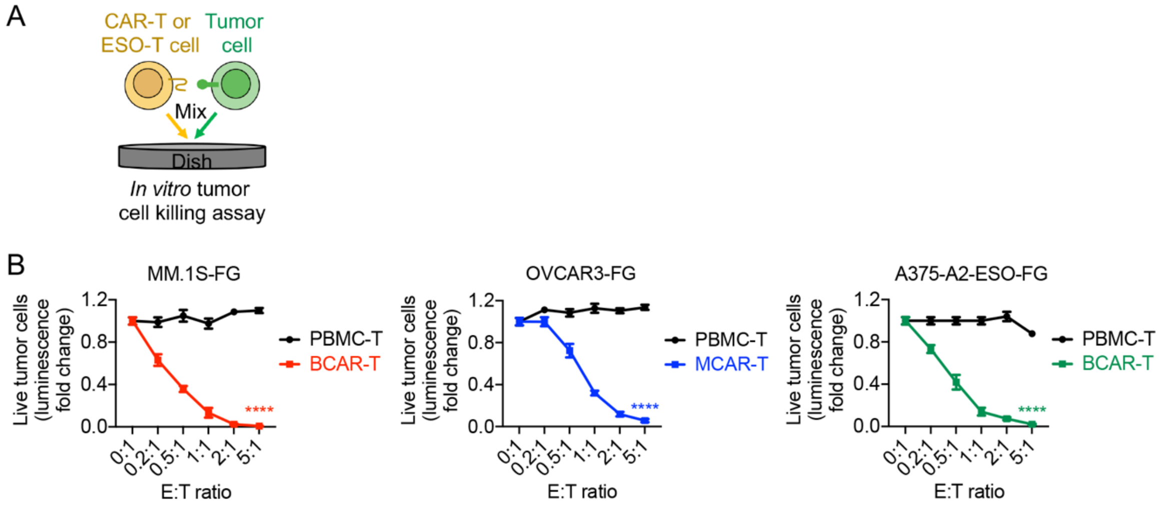

Figure S3. ***In vitro*** **antitumor efficacy of CAR-T and ESO-T cells. Related to Figure 3.**
(A) Experimental design.
(B) Tumor killing data at 24 h (n = 4).
Representative of 3 experiments. Data are presented as the mean ± SEM. ****p < 0.0001 by Student's *t* test.
